# P01.08. Acupuncture alleviates affective dimension of pain in a rat model of inflammatory hyperalgesia

**DOI:** 10.1186/1472-6882-12-S1-P8

**Published:** 2012-06-12

**Authors:** L Lao, Y Zhang, X Shen, A Li, J Xin, B Berman, M Tan, K Ren, R Zhang

**Affiliations:** 1 Center for Integrative Medicine, Baltimore, USA; 2 Shanghai University of Traditional Chinese Medicine, Shanghai, China; 3 Dept. of Neural and Pain Sciences, Dental Sch., Univ. of Maryland, Baltimore, USA

## Purpose

Pain has both sensory and affective dimensions. Previous studies demonstrate that electroacupuncture (EA) alleviates the sensory dimension. This study was to determine whether EA inhibits affective pain and, if so, to study the possibility that rostral anterior cingulate cortex (rACC) opioids underlie this effect.

## Methods

An inflammatory pain rat model, produced by a complete Freund adjuvant (CFA) injection into the hind paw of each rat, was combined with a conditioned place avoidance (CPA) test. On day one, time spent by the rats in each of two distinct compartments during a 10-min period was recorded. On day two, each rat explored one of the conditioning compartments for 30 min. Two hours after the CFA injection, each rat was allowed to explore the second compartment for 30 min. On day three, the time spent by the rats in each compartment was recorded again.

## Results

The rats showed place aversion (i.e. affective pain) by spending less time in a pain-paired compartment after conditioning than during a preconditioning test. An analgesic dose of intrathecal morphine inhibited acquisition of the aversive response but had no effect on display of an established one; systemic non-analgesic morphine inhibited acquisition and display of the affective reaction. This suggests that affective pain is underpinned by mechanisms different from those governing the sensory dimension. The effect of EA was evaluated before pain-paired conditioning and before a post-conditioning test. Rats given EA showed no aversion to the pain-paired compartment, indicating that the treatment inhibits acquisition and display of affect. Mu and kappa opioid receptor antagonists (CTOP and nor-BNI) blocked EA inhibition of acquisition and display, respectively, of the affective dimension.

## Conclusion

EA activates opioid receptors in the rACC to inhibit affective pain and EA may be an effective therapy for both the sensory and the affective dimensions of pain.

